# Multilevel Thresholding Method Based on Electromagnetism for Accurate Brain MRI Segmentation to Detect White Matter, Gray Matter, and CSF

**DOI:** 10.1155/2017/6783209

**Published:** 2017-11-09

**Authors:** G. Sandhya, Giri Babu Kande, T. Satya Savithri

**Affiliations:** ^1^Department of ECE, VNITSW, Guntur, Andhra Pradesh, India; ^2^Department of ECE, VVIT, Guntur, Andhra Pradesh, India; ^3^Department of ECE, JNTUCE, Hyderabad, Telangana, India

## Abstract

This work explains an advanced and accurate brain MRI segmentation method. MR brain image segmentation is to know the anatomical structure, to identify the abnormalities, and to detect various tissues which help in treatment planning prior to radiation therapy. This proposed technique is a Multilevel Thresholding (MT) method based on the phenomenon of Electromagnetism and it segments the image into three tissues such as White Matter (WM), Gray Matter (GM), and CSF. The approach incorporates skull stripping and filtering using anisotropic diffusion filter in the preprocessing stage. This thresholding method uses the force of attraction-repulsion between the charged particles to increase the population. It is the combination of Electromagnetism-Like optimization algorithm with the Otsu and Kapur objective functions. The results obtained by using the proposed method are compared with the ground-truth images and have given best values for the measures sensitivity, specificity, and segmentation accuracy. The results using 10 MR brain images proved that the proposed method has accurately segmented the three brain tissues compared to the existing segmentation methods such as* K*-means, fuzzy* C*-means, OTSU MT, Particle Swarm Optimization (PSO), Bacterial Foraging Algorithm (BFA), Genetic Algorithm (GA), and Fuzzy Local Gaussian Mixture Model (FLGMM).

## 1. Introduction

The present use of neuroimaging procedures allows the scientists and specialists to detect and distinguish various activities and the complications inside the human brain without using any intrusive neurosurgery. Though there are many medical imaging techniques, Magnetic Resonance Imaging is the best imaging technique due to no radiation exposure hence no side effects and it is highly accurate in detecting abnormalities in the internal structures of human organs. The structure of the brain is complex and its tissue segmentation is very crucial to visualize and quantify various brain disorders.

Noise is the main parameter that affects the medical image segmentation. Images can be denoised by using various spatial filters like the low-pass, median, adaptive filter, and so forth. But these filters blur the sharp lines or edges, may respect the edges but the resolution gets decreased by abolishing fine details, and may generate artifacts [1–3]. To overcome the drawbacks of spatial filters Perona and Malik proposed anisotropic diffusion filter [4, 5] which has the properties of (a) sharpening the discontinuities, (b) preserving detailed structures and object boundaries so loss information is minimized, and (c) removing noise in homogeneous regions.

### 1.1. State-of-the-Art Review

A wide range of algorithms has been proposed for the automatic segmentation MR images [6–9]. Image segmentation is a fundamental task in the process of image analysis. Segmentation divides the total image into small regions based on the intensity distribution of the pixels. Thresholding [10–12] is a simple technique for the image segmentation. It separates the object in an image from its background by using an appropriate gray-level value called the threshold. Choosing threshold is very difficult in brain image as the intensity distribution in it is complex. Region-growing [13–15],* K*-means clustering [16, 17], Expectation Maximization (EM) [18, 19], and fuzzy* C*-means (FCM) [20, 21] are the widely used techniques for the medical image segmentation and are the extensions to thresholding. The main drawbacks of these methods are long computational time, sensitivity to noise and sensitivity to the initial guess, very slow convergence, and having no global solution.

Otsu and Kapur proposed two methods for thresholding [22–25]. The first approach maximizes the between-class variance and the other maximizes the entropy between the classes to find the homogeneity. These are reliable for bilevel thresholding [26]. When these algorithms proposed by Otsu and Kapur are used to segment the images of complex intensity distributions which can be effectively segmented by Multilevel Thresholding (MT), the algorithms will extensively search for multiple thresholds which is computationally tedious and the computation time depends on the complexity of the image. Many techniques were developed to reduce the computation time such as [27–29] that are specifically designed to accelerate the computation of objective function, [30–32] that involve Sequential Dichotomization, [33] that is based on an iterative process, and [34] that consists of some Metaheuristic Optimization Techniques. There are methods to solve the problem of determining threshold number in MT process. In [27, 30] multiphase level set method and a new criterion for Multilevel Thresholding are specified in which the optimal threshold number is found by optimizing a cost function. Genetic Algorithm (GA) is combined with wavelet transform [35, 36] to reduce the time.

Evolutionary optimized MT methods are best in terms of speed, accuracy, and robustness compared to classical MT techniques. In [37], various evolutionary approaches such as Differential Evolution (DE), Tabu Search (TS), and Simulated Annealing (SA) are discussed to solve the limitations of Otsu's and Kapur's approaches for MT. In [37, 38], Genetic Algorithms (GAs) based methodologies are utilized for the segmentation of multiclasses. Particle Swarm Optimization (PSO) [39] has been considered for MT, to maximize Otsu's objective function. Other methods [26, 39–43] such as Artificial Bee Colony (ABC), Bacterial Foraging Algorithm (BFA), and Fuzzy Local Gaussian Mixture Model (FLGMM) were developed for the brain image segmentation.

As the proposed method performance is compared with some of the state-of-the-art methods such as *K*-Means, FCM, Otsu MT, PSO, BFA, GA, and FLGMM these are summarized in the following section.

### 1.2. *K*-Means Clustering

*K*-means clustering [16, 17] is an extensively used technique for the image segmentation. This is an iterative method that classifies the pixels of a given image into *k* distinct clusters by converging to a local minimum. Hence the clusters generated are independent and compact. The algorithm comprises two phases. In the first phase, *k* centers are selected randomly, by choosing the value of *k* in advance. The other phase is to bring every pixel to the closest center. Euclidean distance is the generally used metric to measure the distance between each pixel and the centers of clusters. Early grouping is being done when all the pixels are included in different clusters. Now *k* new centroids are refigured for every cluster. In the wake of having these *k* new centroids, another binding must be done between the same group of pixels and the closest new center. This is an iterative process during which the location of *k* centers will change repeatedly until no more changes are done or, in another way, this iterative procedure continues until the criterion function converges to the minimum.

*K*-means is fast, robust, relatively efficient, and easier to understand, and it gives excellent result when data is well separated. The main drawbacks of the *k*-means are as follows: it requires prior specification of the cluster center number, it is unable to divide highly overlapping data, the same data with different representations gives different results, it is sensitive to noise, and the algorithm does not work for the nonlinear type of data.

### 1.3. Fuzzy* C*-Means Clustering

The fuzzy *C*-means algorithm [20, 21] is widely preferred for the medical image segmentation due to its flexibility of allowing pixels to have a place in multiple classes with different degrees of membership and, compared to other clustering methods, it retains more pixel information in the given image. FCM method partitions the pixels of a given image into “*c*” fuzzy clusters regarding some criteria. Different similarity measures such as connectivity, distance, and intensity are used to separate the pixels. In this work, brain images are segmented into three clusters specifically White Matter, Gray Matter, and CSF based on the feature values.

The algorithm is based on the minimization of the objective function:

(1) $$\begin{array}{c} F\left(\;U,c_{1},c_{2},c_{3},\ldots ,c_{c}\;\right)=\overset{c}{\underset{i=1}{\sum }}F_{i}=\overset{c}{\underset{i=1}{\sum }}\overset{n}{\underset{i=1}{\sum }}\mu_{ij}^{m}d_{ij}^{2}. \end{array}$$

*μ*_*ij*_ is the membership value and it is in the range [0,1], *c*_*i*_ is the centroid of the *i*th cluster, *d*_*ij*_ is the Euclidian Distance between *i*th cluster centroid *c*_*i*_ and *j*th data point, and *m* is a weighting exponent in the range [1, *∞*].

Fuzzy clustering of the data samples is carried out through an iterative optimization of the above objective function by updating the membership value *μ*_*ij*_ and the cluster centers *c*_*i*_ by

(2) μij=1∑k=1cdij/dkj2/m−1,ci=∑j=1nμij.mxj∑j=1nμijm.

The major operational drawbacks of FCM are as follows: it is time-consuming and hence it achieve the stabilization condition after a long time, and it does not consider any local or spatial information of the image, and hence it is easily affected by noise and other imaging artifacts.

### 1.4. Otsu Thresholding

Bilevel thresholding can be used to segment the simple images whose object has clear boundaries. But, for the segmentation of complicated images, Multilevel Thresholding (MT) is required. Otsu bilevel thresholding is a well-known nonparametric technique for the segmentation of medical images and it deals with discriminate analysis [22–24]. The value of gray-level at which between-class variance is maximum or within-class variance is minimum is selected as the threshold. This bilevel thresholding divides the pixels of a given image into two separate classes *Cl*_0_ and *Cl*_1_, and it belongs to objects and background at the gray-level th; that is, *Cl*_0_ = {0,1, 2,…, th} and *Cl*_1_ = {th + 1, th + 2,…, *L* − 1}. Let *σ*_*w*_^2^, *σ*_*B*_^2^, and *σ*_*T*_^2^ be the within-class variance, between-class variance, and the total variance, respectively. By minimizing one of the below criterion functions with respect to th an optimal value for the threshold can be found. The criterion functions are

(3) λ=σB2σw2,η=σB2σT2,κ=σT2σw2where  σT2=∑i=0L−11−μT2Pi,  μT=∑i=0L−1iPi,  σB2=W0W1μ0μ12,  W0=∑i=0tPi,  W1=1−W0,  μ1=μT−μt1−w0,  μ0=μtW0,  μt=∑i=0tiPi,  Pi=nin,

and here *n*_*i*_ is the number of pixels with *i*th gray-level, *n* is the total number of pixels in the given image, and *n* = ∑_*i*=0_^*L*−1^*n*_*i*_.  *P*_*i*_ is the probability of occurrence of *i*th gray-level. *W*_0_, *W*_1_ are the areas occupied by the two classes *Cl*_0_ and *Cl*_1_, respectively, and *μ*_0_, *μ*_1_ are the mean values of the classes *Cl*_0_ and *Cl*_1_, respectively.

Among the criterion functions, *η* is minimum. Hence, the optimal threshold th = argmin⁡*η*. The maximum estimate of *η*, designated as *η*^*∗*^, is used to evaluate the amount of separability of classes *Cl*_0_ and *Cl*_1_. It is very significant as it does not vary under affine transformations of the gray-level scale.

Otsu bilevel thresholding can be extended to MT and is direct by virtue of the discriminant criterion. For instance, on account of three-level thresholding, two thresholds are defined as 1 ≤ th_1_ < th_2_ < *L* for separating three classes, *Cl*_0_ for {0,1, 2,…, th_1_}, *Cl*_1_ for {th_1_ + 1, th_1_ + 2, th_2_}, and *Cl*_2_ for {th_2_ + 1, th_2_ + 2,…, *L*}. The between-class variance *σ*_*B*_^2^ is a function of th_1_ and th_2_, and the optimal thresholds th_1_^*∗*^ and th_2_^*∗*^ can be found by maximizing the function *σ*_*B*_^2^, where *σ*_*B*_^2^(th_1_^*∗*^, th_2_^*∗*^) = max_1≤th_1_<*th*_2_<*L*_⁡*σ*_*B*_^2^(th_1_, th_2_).

The main drawback of the Otsu is the following: as the number of segments to be divided increases, the selected thresholds become less accurate. This is simple and effective for two-level and three-level thresholding, which can be applied to almost all applications.

### 1.5. Particle Swarm Optimization (PSO)

PSO [39] is a population-based stochastic optimization process. The method searches for a solution by altering the directions of individual vectors, termed as “particles.” The initial location of the particles is chosen randomly from the search area Ω. For every iteration, a velocity is assigned to every particle in Ω and it gets updated by the best value that the particle has visited. Then, using the updated velocity in iteration the position of every particle is updated. The performance of the particle is assessed by its fitness function value. At every iteration, the values of the best positions visited by the particle and their companions are saved as personal and population observations by which every particle will converge to the optimal solution. Thus, PSO has quick convergence compared to the other population-based methods such as DE or GA.

In the *N*-dimensional search space, the position vector of the *i*th particle is defined as *X*_*i*_ = (*x*_*i*1_, *x*_*i*2_,…, *x*_*iN*_) and its velocity vector as *V*_*i*_ = (*v*_*i*1_, *v*_*i*2_,…, *v*_*iN*_). According to a predefined fitness function, if *P*_*b*_ = (*p*_*b*1_, *p*_*b*2_,…, *p*_*bN*_) and *P*_*f*_ = (*p*_*f*1_, *p*_*f*2_,…, *p*_*fN*_) are assumed as the best position of each particle and the fittest particle for an iteration *t*, respectively, then, the new position and velocities of the particles for the next fitness function are calculated as

(4) Vit+1=αVit+k1rand1∗Pb−Xi+k2rand2∗Pf−Xi,

(5) Xit+1=Xit+Vit+1,

where *k*_1_ and *k*_2_ are positive constants and rand_1_ and rand_2_ are two random functions uniformly distributed in the interval (0, 1). The variable *α* representing the inertia weight causes the convergence of the algorithm. The PSO algorithm can be surely converged if each particle must converge to its local attractor

(6) Q=Q1,Q2,…,QN,where  QN=rand∗pbN+1−rand∗pfN.

According to (4), all the particles are greatly influenced by *P*_*b*_ and *P*_*f*_. If the best particle reaches a local optimum, then all the remaining particles will fast converge to the location of the final best particle. Hence, in the PSO global optimum of the fitness function is not guaranteed which is called premature.

A globally optimal solution is considered as a feasible solution whose objective value is better than other feasible solutions. For locally optimal solution no better feasible solutions can be found in the immediate neighborhood of the given solution. Subsequently, if the algorithm loses the diversity at early iterations it may get trapped into local optima, and it implies that the population turns out to be exceptionally uniform too soon. Despite the fact that PSO finds good solutions faster than other evolutionary algorithms it generally can not enhance the quality of the solution as the number of iterations is improved. The solution of the global best is improved when the swarm is iterated. It could happen that all particles being influenced by the global best eventually approach the global best and from there on the fitness never improves despite however many runs the PSO is iterated thereafter. The particles also move in the search space in close proximity to the global best and not exploring the rest of the search space. This is called premature convergence.

### 1.6. Bacterial Foraging Algorithm (BFA)

BFA optimization strategies are methods for locating, handling, and ingesting food. Natural selection eliminates the animals with poor foraging methodologies. This encourages the propagation of qualities of the best foraging methods. After so many generations, the poor foraging strategies are either wiped out or upgraded into better ones. A foraging animal tries to maximize the energy intake per unit time spent on foraging within its environmental and physiological constraints. The* E. coli* bacteria, present in human intestine, follow foraging behavior, which consists of processes of chemotaxis, swarming, reproduction, and elimination or dispersal. In [42, 44] this evolutionary technique was modeled as an effective optimization tool. 

*Chemotaxis*. The bacterial movement of swimming and tumbling in presence of attractant and repellent chemicals from other bacteria is called chemotaxis. A chemotactic step is a tumble followed by a tumble or run. After defining a unit length random direction the chemotaxis can be modeled as

(7) $$\begin{array}{c} X^{i}\left(\;j+1,k,l\;\right)=X^{i}\left(\;j,k,l\;\right)+S\left(\;i\;\right)\lambda \left(\;j\;\right), \end{array}$$

where *X*^*i*^(*j*, *k*, *l*) is the *i*th bacterium at *j*th chemotactic, *k*th reproductive, and *l*th elimination or dispersal event. *S*(*i*) is the step size in the direction of movement specified by tumble called the run length unit.

*Swarming*. Bacterium which reaches a good food source produces chemical attractant to invite other bacteria to swarm together. While swarming, they maintain a minimum distance between any two bacteria by secreting chemical repellent. Swarming is represented mathematically as

(8) JccX,Pj,k,l=∑i=1TJiccX,Xij,k,l=∑i=1T−dattractexp⁡−wattract∑n=1mXn−Xi2+∑i=1Threpellantexp⁡−wrepellant∑n=1mXn−Xi2⁡

and here *Jcc*(*X*, *P*(*j*, *k*, *l*)) is the value of the cost function to be added to the optimized actual cost function to simulate the swarming behavior, *T* is the total number of bacteria, *m* is the number of parameters to be optimized, and *d*_attract_, *w*_attract_, *w*_repellant_, and *h*_repellant_ are the coefficients to be chosen properly. 

*Reproduction*. After completion of *N*_*c*_ chemotactic steps, a reproductive step follows. Health of *i*th bacterium is determined as

(9) $$\begin{array}{c} J_{health}^{i}=\overset{N_{c}}{\underset{i=1}{\sum }}J_{sw}\left(\;i,j,k,l\;\right) \end{array}$$

Then, in the descending order of their health, the bacteria are sorted. The least healthy bacteria die and the other healthier bacteria take part in reproduction. In reproduction, each healthy bacterium splits into two bacteria each containing identical parameters as that of the parent keeping the population of the bacteria constant. 

*Elimination and Dispersal*. The bacterial population in a habitat may change gradually due to the constraint of food or, suddenly, due to environmental or any other factor. Every bacterium in a region might be killed or some might be scattered into a new location. It may have the possibility of annihilating chemotactic progress, but it also has the ability to help chemotaxis, since dispersal event may put the bacteria to near-good food sources.

### 1.7. Genetic Algorithm (GA)

GAs [45, 46] are effective, flexible, and powerful optimization procedures governed by the standards of evolution and natural genetics. GAs have implied parallelism. This algorithm begins with the chromosomal modeling of a set of parameters that will be coded as a limited size string over letters in order of limited length. An arrangement of the chromosomes in a generation is known as population, the measure of which might be consistent or may change starting with one generation and then onto another. In the initially defined population, the chromosomes are either produced randomly or utilizing domain-specific data information. The fitness function is designed, such that the strings or possible solutions that have high fitness values are characterized as best points in the feasible search region. This is known as the payoff information that is used by the GAs to search for probable solutions. During reproduction individual strings are replicated into a temporary new population called the mating pool, to convey genetic operations. The sum of copies received by an individual corresponds to the fitness value and these are used for the next generation. In general, the chromosome which is retained in the population till the final generation is treated as the best chromosome. Exchange of data between arbitrarily chosen parent chromosomes by combining details of their genetic information is treated as crossover. The efficiency of GAs mainly depends on the coding-crossover strategy. Chromosome's genetic structure can be altered by the process of mutation which is used to bring the genetic diversity into the population.

There are many difficulties and issues in using GAs for image segmentation. The strategy of encoding should be confirmed to the Building Block Hypothesis; otherwise GA gives the poor result. The performance of GAs depends on the design of fitness function in such a way to reduce the computation time, choice of various genetic operators, termination criteria, and methods of keeping off premature convergence.

### 1.8. Fuzzy Local Gaussian Mixture Model (FLGMM)

In the case of ordinary GMM, it has been assumed that intensities of a region are sampled individually from an identical Gaussian distribution function. This stochastic assumption is not suitable for MR brain images because of the presence of bias field. However, in the recently proposed FLGMM [43] algorithm for MR brain image segmentation, the bias field is expressed as a slowly varying quantity and it can be overlooked inside a small window. The objective function of FLGMM is obtained by integrating the Gaussian Mixture Model (GMM) weighted energy function over the image. The function consists of a truncated Gaussian kernel to establish the spatial constraints and fuzzy memberships to balance the contribution of GMM for segmentation.

In the process of FLGMM, based on the fuzzy* C*-means model of the images with intensity inhomogeneity the local intensity clustering property or the image partitions are derived from the input image. A local objective function is formulated for the given image. Minimization of the energy function is performed by defining individual membership functions locally for each cluster by assuming that it satisfies the Gaussian Mixture Model. A bias-field equivalent to the intensity inhomogeneity is generated. Thus energy minimization generates a homogenous image which is termed as the intensity inhomogeneity corrected image.

The remaining part of the paper is methodized as follows.  Section 2 describes the materials and methods utilized in this work.  Section 3 consists of experimental results and discussion.  Section 4 is the brief concluding section.

## 2. Materials and Methods

**Figure 1:**
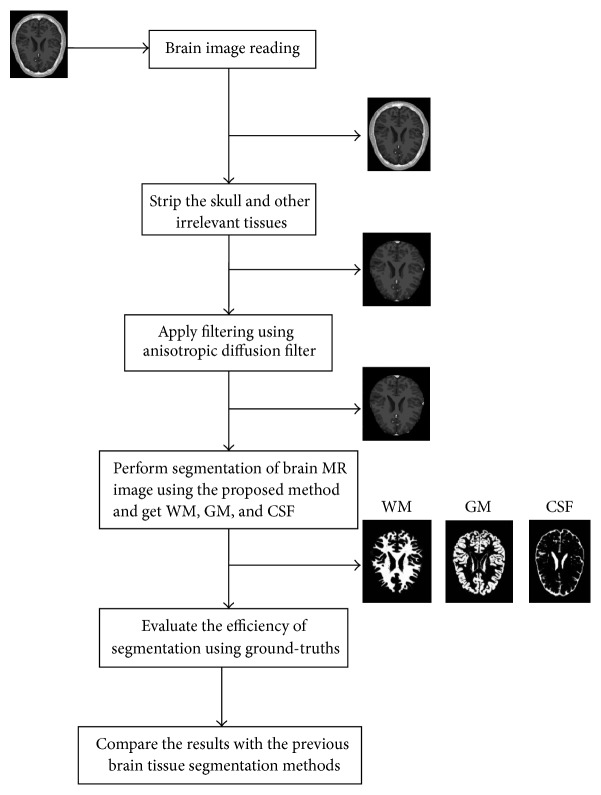
Flow diagram of the proposed method.

The present section describes the materials and methods used in this work. The overall algorithm is presented like a flow diagram in Figure 1. The stages involved in the implementation of the algorithm are explained in the subsections. The proposed method is implemented in MATLAB.

### 2.1. Brain MR Images

The MR images used in this method are downloaded from the BrainWeb [47] database. These are the T1-weighted brain images of 10 different subjects.

### 2.2. Preprocessing

In the preprocessing stage, two issues are considered. First is the skull stripping and second is improving SNR by using anisotropic diffusion filter.

#### 2.2.1. Skull Stripping

**Figure 2:**
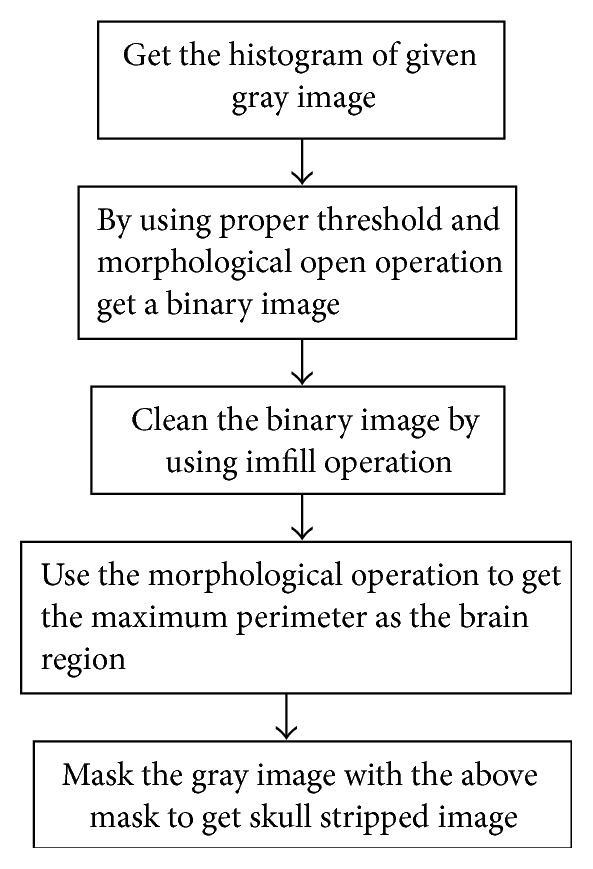
Skull stripping algorithm.

Skull stripping is an essential phenomenon to study the neuroimaging data. Numerous applications, like cortical surface reconstruction, presurgical planning, and brain morphometry, depend on accurate segmentation of brain region from nonbrain tissues such as skin, skull, and eyeballs. The algorithm for skull stripping is presented in Figure 2. This is based on brain anatomy and image intensity. It combines the estimation of adaptive threshold and morphological operations, to give better results. This is an automatic method which does not need any user interaction to choose any parameters for the brain matter extraction. Some of the existing techniques for skull stripping such as AFNI, FSL, and SPM require some parameter adjustments to get better results for various brain image data sets. Jaccard Similarity Coefficient (JSC) is used to compare the performance of the proposed skull stripping method with the above-mentioned methods. JSC measures the similarity between the skull stripped image and the ground-truth image and is defined as the size of the overlapping area of the two images divided by the size of the union of the two images.

(10) $$\begin{array}{c} J\left(\;A,B\;\right)=\frac{\left|A\cap B\right|}{\left|A\cup B\right|}. \end{array}$$

The values of JSC are tabulated in Table 1. Exploratory outcomes guarantee that the proposed skull stripping process is appropriate for both synthetic and real images, though the real images are of low contrast. It works very well even for the brain images where the previously mentioned techniques fail.

#### 2.2.2. Filtering

In this proposed work noise presented in the MR images is decreased by using anisotropic diffusion filter designed by Perona and Malik [5]. This algorithm decreases the noise in the image by revising it through PDE. It smoothens the textures in an image by the law of diffusion on the intensities of pixels. Diffusion across edges is prevented by a threshold function; therefore it respects the edges presented in the image.

### 2.3. Segmentation

MR images of the brain can be segmented by using MT. The existing MT techniques are computationally extravagant as they intensely inquire the best values for the optimization of an objective function. This work uses an advanced MT technique named as Electromagnetism-Optimization (EMO) algorithm [48]. This is formulated using the phenomenon of the force of “attraction-repulsion” between charges to make the associates of the population. The algorithm incorporates the excellent search capabilities of the objective functions proposed by Otsu and Kapur. This considers arbitrary samples against a feasible search area in the histogram of the brain image. These samples develop the particles in the process of EMO and their quality is measured by the objective functions of Otsu or Kapur. These values of the objective functions guide the candidate solutions to evolve through the operators of EMO until an optimal solution is reached. This process develops a segmentation method based on MT which has the ability to identify different threshold values for the segmentation of MR brain image in a minimum number of iterations.

Different to the other metaheuristics approaches such as Differential Evolution (DE), Genetic Algorithm (GA), Artificial Bee Colony (ABC), and Artificial Immune System (AIS), where there is exchange of information between the members of population, in EMO each particle is affected by others within the population like in heuristics approaches such as Particle Swarm Optimization (PSO) and Ant Colony Optimization (ACO). Though it has few characteristics similar to other evolutionary optimized approaches [49], it exhibits a better accuracy with respect to its optimal parameters, optimal convergence [50], and decreased computation time compared to other methods for brain image segmentation. This has been utilized in communications, optimization in circuits, control systems, training of neural networks, and image processing.

#### 2.3.1. Electromagnetism-Optimization Algorithm (EMO)

This method is designed to propose a global solution for a nonlinear optimization problem defined in the following form:

(11) maximize fx,where  x=x1,…,xn∈Rn subject  to x∈X,

where *f*: *ℜ*^*n*^ is a nonlinear function and *X* = {  *x* ∈ *ℜ*^*n*^∣*l*_*i*_ ≤ *x*_*i*_ ≤ *u*_*i*_, *i* = 1,…, *n*} represents bounded feasible search space limited by the lower limit *l*_*i*_ and the upper limit *u*_*i*_. The algorithm uses* N*,* n*-dimensional points *x*_*i*,*k*_, to represent the population for analyzing the set *X*, and* k *is the iteration number. The original population being *k* = 1 is *S*_*k*_ = {*x*_1,*k*_, *x*_2,*k*_,…, *x*_*N*,*k*_}, are uniformly distributed samples taken from *X*. As the members of population are changed according to* k* the set of the population at the *k*th iteration is denoted by *S*_*k*_. After defining *S*_*k*_, the algorithm extends the process iteratively until a terminating condition is reached. During the first step of iteration every point in *S*_*k*_ shifts to another location using the mechanism of attraction-repulsion [51], and, in the next step, these already displaced points are further displaced locally with a local search and become the members of the set *S*_*k*+1_ in the (*k* + 1)th iteration. It is the responsibility of Electromagnetism and the local search to drive the members of population *x*_*i*,*k*_ of the set *S*_*k*_ to the proximity of the global optimization.

Analogous to the theory of Electromagnetism between charges, every point *x*_*i*,*k*_ ∈ *S*_*k*_ in the region *X* is treated like a charged particle and the charge associated with it is assumed as the value of objective function. Particles with the larger value of the objective function have associated with more charge than the particles with poor objective function. In the process of EMO the points with more charge exhibit force of attraction on the other points in *S*_*k*_, and the points with less charge will exhibit repulsion. Finally, the force vector *F*_*i*_^*k*^, exerted on *i*th point *x*_*i*,*k*_, is computed by adding the forces on all charged points and each point *x*_*i*,*k*_ ∈ *S*_*k*_ is moved along the total force direction to the destination position *y*_*i*,*k*_. A simple local search is made to examine the proximity of each *y*_*i*,*k*_ by *y*_*i*,*k*_ to *z*_*i*,*k*_. The members, of the (*k* + 1)th iteration, that is, *x*_*i*,*k*+1_ ∈ *S*_*k*+1_, can be found using

(12) $$\begin{array}{c} x_{i,k+1}=\left\{\begin{array}{cc} y_{i,k} & \text{if }f\left(y_{i,k}\right)\leq f\left(z_{i,k}\right)\\ z_{i,k} & \text{otherwise.} \end{array}\right. \end{array}$$

The following stepwise algorithm shows the process of EMO.

**Step 1 .**

Get the input parameters *k*_max_, *k*_local_, *δ*, and *N* where *k*_max_ is the maximal number of iterations. *n* × *k*_local_ are the maximum number of locations *z*_*i*,*k*_, inside *X* within a *λ* distance from*y*_*i*,*k*_ for each *i* dimension.

**Step 2 .**

For *k* = 1, the points *x*_*i*,*k*_ are selected uniformly in *X*, that is, *x*_*i*,1_ as uni(*X*), where uni is the uniform distribution function. The values of the objective function *f*(*x*_*i*,*k*_) are calculated and the best point *x*_*k*_^*B*^ which produces the maximum value of *f*(*x*_*i*,*k*_) in the space *S*_*k*_ is identified as

(13) $$\begin{array}{c} x_{k}^{B}=\underset{x_{i,k}\in S_{k}}{argmax}\left\{\;f\left(\;x_{i,k}\;\right)\;\right\}. \end{array}$$

**Step 3 .**

Each point *x*_*i*,*k*_ is assigned with a charge *q*_*i*,*k*_. The charge *q*_*i*,*k*_ depends on the objective function *f*(*x*_*i*,*k*_). Points with the large value of the objective function have more amount of charge than the points with the poor value of objective function. The charge is calculated as follows:

(14) $$\begin{array}{c} q_{i,k}=\left[\;-n\frac{f\left(x_{i,k}\right)-f\left(x_{k}^{B}\right)}{\sum_{j=1}^{N}f\left(x_{i,k}\right)-f\left(x_{k}^{B}\right)}\;\right]. \end{array}$$

The force *F*_*i*,*j*_^*k*^ between two points *x*_*i*,*k*_ and *y*_*i*,*k*_ is calculated using

(15) Fi,jk=xj,k−xi,kqi,kqj,kxj,k−xi,k2if  fxi,k>fxj,kxi,k−xj,kqi,kqj,kxj,k−xi,k2if  fxi,k≤fxj,k.

The total force *F*_*i*_^*k*^ corresponding to *x*_*i*,*k*_ is

(16) $$\begin{array}{c} F_{i}^{k}=\overset{N}{\underset{j=1,j\neq i}{\sum }}F_{i,j}^{k}. \end{array}$$

**Step 4 .**

Except for *x*_*k*_^*B*^, each point *x*_*i*,*k*_ is moved along the total force *F*_*i*_^*k*^ using

(17) xi,k=xi,k+μFikFik  Range,i=1,2,…,N,  i≠B,

where *μ* = unif(0,1) for each coordinate of *x*_*i*,*k*_.* Range* represents the allowed lower or upper bound.

**Step 5 .**

In the neighborhood *λ* of *y*_*i*,*k*_, a maximum of *k*_local_ points are generated. Generation of local points is continued until either a better *z*_*i*,*k*_ is found or the *n* × *k*_local_ number of iterations is reached.

**Step 6 .**

In the next iteration *x*_*i*,*k*+1_ ∈ *S*_*k*+1_ are selected from *y*_*i*,*k*_  and *z*_*i*,*k*_ using (11), and the best is found using (13).

The EMO algorithm has the analysis for its complete convergence [52]. This algorithm shows the existence of at least one sample of the population *S*_*k*_ which is moving very close to the optimal solution in a single iteration. Hence EMO method can solve the complicated optimization problems in a minimum number of iterations. This is proved in several studies of EMO [53–55] where its less computational cost and its minimum iteration number were compared with other evolutionary algorithms for various engineering related issues.

#### 2.3.2. Formation of *f*_*Otsu*_

Considering the *L* intensity values of a gray scale image, the probability distribution of the intensity levels can be calculated as

(18) Phic=hicNP,∑i=1NPPhic=1,c=1  for  gray  scale  image,

where *i* is a particular intensity level between 0 and *L* − 1, *c* is the component of the image, and NP represents the total number of pixels in the image. The histogram *h*_*i*_^*c*^ is defined as the number of pixels having the *i*th intensity level in component. The histogram is normalized to get the probability distribution *Ph*_*i*_^*c*^. In the case of bilevel thresholding the two classes *C*_1_ and *C*_2_ are defined as

(19) $$\begin{array}{c} C_{1}=\frac{{Ph}_{1}^{c}}{w_{0}^{c}\left(th\right)},\ldots ,\frac{{Ph}_{th}^{c}}{w_{0}^{c}\left(th\right)},\\ \frac{{Ph}_{th+1}^{c}}{w_{0}^{c}\left(th\right)},\ldots ,\frac{{Ph}_{L}^{c}}{w_{0}^{c}\left(th\right)} \end{array}$$

and here *w*_0_(*th*) and *w*_1_(*th*) are the probabilities distributions for the classes *C*_1_ and *C*_2_:

(20) w0cth=∑i=1thPhic,w1cth=∑i=th+1LPhic.

The mean values *μ*_0_^*c*^ and *μ*_1_^*c*^ of the classes and the between-class variance *σ*_*B*_^2*c*^ can be written as

(21) μ0c=∑i=1thiPhicw0cth,μ1c=∑i=th+1LiPhicw1cth,σB2c=σ1c+σ2c,

where *σ*_1_^*c*^ and *σ*_2_^*c*^ are the variances of *C*_1_ and *C*_2_:

(22) σ1c=w0cμ0c+μTc2,σ2c=w1cμ1c+μTc2

and here *μ*_*T*_^*c*^ = *w*_0_^*c*^*μ*_0_^*c*^ + *w*_1_^*c*^*μ*_1_^*c*^ and *w*_0_^*c*^ + *w*_1_^*c*^ = 1. Based on the values of *σ*_1_^*c*^ and *σ*_2_^*c*^ the objective function is defined as follows:

(23) $$\begin{array}{c} f_{otsu}\left(\;th\;\right)=\max\left(\;\sigma_{B}^{2c}\left(\;th\;\right)\;\right),\;0\leq th\leq L-1, \end{array}$$

where *σ*_*B*_^2*c*^(th) is Otsu's variance for a given th value. Hence the optimization problem becomes to find a value of th that can maximize the above objective function.

This bilevel method can be extended for the identification of multiple thresholds. Considering *k* thresholds it is possible to separate the original image into *k* classes as above. Then the objective function *f*_otsu_(th) for multiple thresholds can be written as follows:

(24) fotsuTH=max⁡σB2cTH,0≤thi≤L−1,  i=1,2,3,…,k,

where TH = [th_1_, th_2_, th_3_,…, th_*k*−1_] is a vector containing multiple thresholds and the variances are computed as

(25) $$\begin{array}{c} \sigma_{B}^{2c}=\overset{k}{\underset{i=1}{\sum }}\sigma_{i}^{c}=\overset{k}{\underset{i=1}{\sum }}w_{i}^{c}{\left(\;\mu_{i}^{c}-\mu_{T}^{c}\;\right)}^{2} \end{array}$$

and here *i* represents the specific class and *w*_*i*_^*c*^ and *μ*_*j*_^*c*^ represent the probability of occurrence and the mean of a class, respectively. For Multilevel Thresholding, these values are obtained as

(26) w0cth=∑i=1th1Phic,w1cth=∑i=th1+1th2Phic,…,wk−1cth=∑i=thk+1LPhic

and for the mean values

(27) μ0c=∑i=1th1iPhicw0cth1,μ1c=∑i=th1+1th2iPhicw1cth2,…,μk−1c=∑i=i=thk+1LiPhicwk−1cthk.

#### 2.3.3. Segmentation Using the Proposed Method

The designed segmentation algorithm can be coupled with two distinct objective functions proposed by Otsu and Kapur developing two different image segmentation algorithms. Kapur's convergence is unstable for certain range of thresholds, but Otsu's is the most stable. Hence, in this work, the EMO algorithm is combined with Otsu thresholding. In the proposed approach, image segmentation is described as an optimization problem that is stated as

(28) maximize fotsuth,th=th1,th2,…,thmsubject  to th∈X

*f*_otsu_(th) is the objective function defined above. The term th = [th_1_, th_2_,…, *m*] represents various thresholds. *X* represents a bounded workable region in the interval [0  255] which corresponds to image intensity levels. In the optimization algorithm, every particle uses *m* elements, as decision variables. These variables represent various thresholds used for the segmentation. Therefore, the total population is characterized as

(29) Sk=TH1c,TH2c,…,THNc,THic=th1c,th2c,…,thmcT,

where *k* is the number of iterations, *T* is the transpose operator, and *N* is the population size. The parameter *c* is set to 1 for gray scale images.

#### 2.3.4. Implementation of EMO Algorithm

We evaluated each method's sensitivity to the number of bins in the histogram and found that The stepwise implementation of the algorithms is as follows.

**Step 1 .**

Read the input gray scale image and name it as *I* and set *c* = 1.

**Step 2 .**

Obtain the histogram *h* of the image.

**Step 3 .**

Calculate the probability distribution functions.

**Step 4 .**

Initialize the parameters *k*_max_, *k*_local_, *λ*, *m*, and *N*.

**Step 5 .**

Initialize the population *S*_*k*_^*c*^ having *N* random particles and *m* dimensions.

**Step 6 .**

Find the values of *w*_*i*_^*c*^ and *μ*_*i*_^*c*^. Compute the *S*_*k*_^*c*^ required to find the objective function *F*_Otsu_.

**Step 7 .**

Calculate the amount of charge associated with each particle and also total force vector.

**Step 8 .**

Move the total population *S*_*k*_^*c*^ along the force vector.

**Step 9 .**

Local search is made in the moved population and best elements are selected based on the values of their objective functions.

**Step 10 .**

The iteration number *k* is incremented in 1; if *k* ≥ *k*_max_ the algorithm stops the iteration and jumps to Step  11; otherwise it jumps to Step  7.

**Step 11 .**

Using *F*_Otsu_ choose the particle *x*_*k*_^*B*^^*c*^ that has the best objective function value.

**Step 12 .**

Use the thresholds values contained in *x*_*k*_^*B*^^*c*^ to the entire image for segmentation.

### 2.4. Evaluation

The objective or automatic evaluation of segmentation is very easy and it involves verifying the segmented pixels against a known pixel-wise ground truth. There are mainly three performance parameters for any segmentation process such as sensitivity, specificity, and segmentation accuracy.

*Sensitivity*. It indicates true positivity and it is the probability that a detected or segmented pixel belongs to the particular tissue.

(30) $$\begin{array}{c} Sensitivity=\frac{TP}{TP+FN}. \end{array}$$

*Specificity*. It indicates true negativity and it is the probability that a detected or segmented pixel does not belong to particular tissue but it belongs to the background.

(31) $$\begin{array}{c} Specificity=\frac{TN}{FP+TN}. \end{array}$$

*Segmentation Accuracy*. It indicates the degree to which segmentation algorithm results match with reference or ground truths.

(32) $$\begin{array}{c} \text{Segmentation Accuracy}=\frac{TP+TN}{TP+TN+FP+FN}. \end{array}$$

Here TP indicates “True Positive” which is the number of pixels exactly detected as particular tissue pixels. TN indicates “True Negative” which is the number of pixels exactly detected as not particular tissue pixels. FP indicates “False Positive” which is the number of pixels wrongly detected as particular tissue pixels. FN indicates “False Negative” which is the number of pixels wrongly detected as not particular tissue pixels.

## 3. Experimental Results and Discussion

This section presents the experimental results of the proposed algorithm in detecting various brain tissues and the comparison with other methods. The algorithm is implemented using MATLAB. The MR images of the brain are downloaded from the BrainWeb database. Around 20 different MR images are used for testing the proposed algorithm, but the results of 10 images are presented in the paper. The recommended segmentation process is evaluated using the measures sensitivity, specificity, and segmentation accuracy.

**Figure 3:**
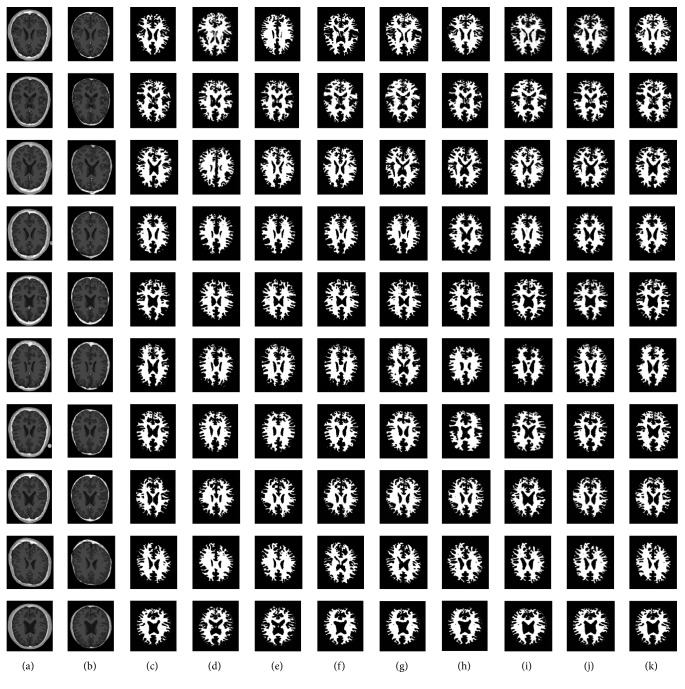
*White Matter Detection.* (a) Brain images; (b) skull stripped images; (c) ground-truth images; (d) results of *K*-means; (e) results of fuzzy *C*-means; (f) results of Otsu MT; (g) results of PSO; (h) results of BFA; (i) results of GA; (j) results of FLGMM; (k) results of the proposed method.

**Figure 4:**
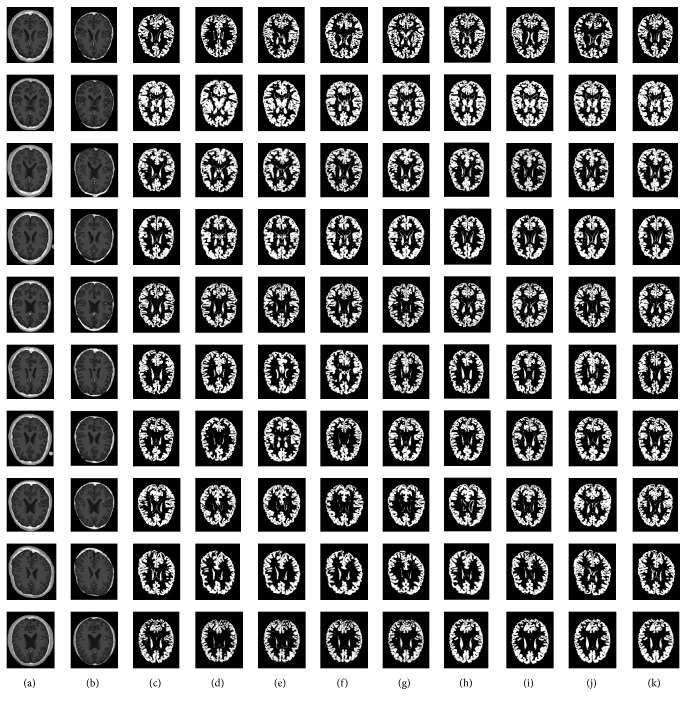
*Gray Matter Detection*. (a) Brain images; (b) skull stripped images; (c) ground-truth images; (d) results of *K*-means; (e) results of fuzzy *C*-means; (f) Results of Otsu MT; (g) results of PSO; (h) results of BFA; (i) results of GA; (j) results of FLGMM; (k) results of the proposed method.

**Figure 5:**
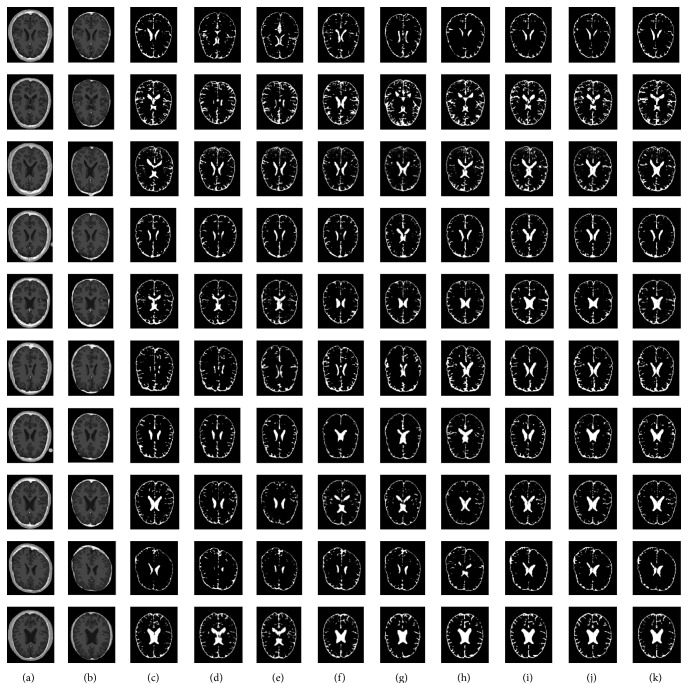
*CSF Detection*. (a) Brain images; (b) skull stripped images; (c) ground-truth images (d) results of *K*-means; (e) results of fuzzy *C*-means; (f) results of Otsu MT; (g) results of PSO; (h) results of BFA; (i) results of GA; (j) results of FLGMM; (k) results of the proposed method.

Figures 3–5 depict the visual results of three types of tissue detection from 10 different MR images of the brain for the proposed and the other methods such as *K*-means [16, 17], fuzzy *C*-means [20, 21], Otsu MT [22–24], Particle Swarm Optimization (PSO) [39], Bacterial Foraging Algorithm (BFA) [42, 44], Genetic Algorithm (GA) [45, 46], and FLGMM [43]. These results show that the proposed method is excellent compared to the others. The method is giving best results even for the images of complex intensity distributions. Results of all the 10 different persons' brain images demonstrate the successful detection of all the tissue types. Positions and sizes of all the tissues are detected correctly and all the segmentation performance measures are quite high for the proposed method. In future work, to increase the performance of segmentation additional features such as prior knowledge, shape, and models can be used during the segmentation.

Tables 2–4 consist of the performance parameters of the proposed segmentation algorithm and the other existing methods in detecting White Matter (WM), Gray Matter (GM), and Cerebral Spinal Fluid (CSF), respectively. It came to be known from the tables that the performance of the algorithm in segmenting the tissues-wise WM, GM, and CSF is quite high. The values of three performance parameters sensitivity, specificity, and segmentation accuracy are almost high for all the 10 images. Hence the overall results show that the method performs well in segmentation compared to the previous methods.

## 4. Conclusions

This paper proposed an excellent and innovative Multilevel Thresholding method to segment different tissues like White Matter (WM), Gray Matter (GM), and CSF from MRIs of the brain. Segmentation of WM and GM and CSF segmentation of brain image are vital in identifying disorders and treatment planning in the field of medicine. This method outperforms well in segmenting all tissues. This method uses the histogram and morphological operations for the skull stripping. Anisotropic diffusion filtering is used in preprocessing to eliminate the noise and also to smoothen the image. An Electromagnetism-Like algorithm which depends on the phenomenon of “attraction-repulsion” between the charges is used for the segmentation. This approach is a combination of effective search potentials of “attraction-repulsion” algorithm with the objective function of popular Otsu MT method.

Different to other algorithms, EMO exhibits interesting search capabilities whereas it maintains a low computational overhead. The constraints of EMO are as follows: if the value of objective function reaches a very high value, the fraction value in the equation of charge (see (8)) becomes very small and creates an overflow problem to find the charge. This can be refrained by assuming a high floating point value for the points having a very high value of objective function. Overflow problem can also occur if the separation between the two points is much nearer to zero and it can be avoided by maintaining a minimum separation between the points based on the word length of the processor. When the force acting on the charged particles discards some parts of the feasible search space, premature convergence may occur which leads to the wrong result. This can be avoided by perturbing the present population so that no less than one point among all the points will have an opportunity to move to the discarded parts of the region. This point will be considered as the best point called “perturbed point” and the force is calculated by taking this perturbed point into account.

With regard to evaluating the performance of the proposed approach, the metrics like sensitivity, specificity, and segmentation accuracy are used, taking into account the similarity between the segmented image and the ground truth. The proposed approach is carried out on 10 different MRI images of the brain which are downloaded from the BrainWeb database.

The recommended approach has been compared with other segmentation algorithms such as Otsu MT [22–24], *K*-means [16, 17], fuzzy *C*-means [20, 21], Particle Swarm Optimization (PSO) [39], Bacterial Foraging Algorithm (BFA) [42], Genetic Algorithm (GA) [36], and Fuzzy Local Gaussian Mixture Model [43]. The experimental results proved the outstanding performance of the proposed algorithm compared to the other existing methods. The performance of the proposed approach in detecting various tissues can be increased by designing an effective objective function. This soft tissues detection of the brain MR image is very important for surgical planning and to find and diagnose neurological diseases.

## Figures and Tables

**Table 1:** Comparison of skull stripping Algorithms.

MRI	AFNI	FSL	SPM	Proposed
(1)	.714	.814	.658	.862
(2)	.654	.698	.729	.756
(3)	.574	.625	.662	.789
(4)	.784	.865	.789	.874
(5)	.678	.695	.628	.685
(6)	.814	.802	.798	.821
(7)	.724	.765	.745	.753
(8)	.814	.825	.874	.832
(9)	.698	.627	.587	.689
(10)	.712	.698	.598	.701

**Table 2:** Performance measures for the proposed method and the existing methods for segmenting White Matter (WM) from 10 different MR images.

Algorithm	Sensitivity										
	MRI 1	MRI 2	MRI 3	MRI 4	MRI 5	MRI 6	MRI 7	MRI8	MRI 9	MRI 10	Avg
*K*-means	85.54	86.04	82.50	81.29	80.78	80.78	81.14	82.14	80.26	83.20	82.36
FCM	86.78	87.94	84.00	83.42	82.18	82.58	83.42	84.00	90.94	80.78	84.60
OTSU	87.24	89.21	89.06	91.72	89.62	89.62	87.72	88.06	88.21	84.24	88.47
PSO	87.98	89.35	90.25	91.25	88.35	89.96	87.26	89.21	85.25	87.25	88.61
BFA	89.25	89.87	93.56	92.35	89.89	90.35	89.23	89.36	89.25	94.32	90.74
GA	90.56	90.28	93.25	93.25	90.25	92.35	92.35	93.25	90.25	95.26	92.10
FLGMM	94.25	91.23	95.35	92.37	92.35	93.25	93.25	94.25	91.25	96.25	93.38
Proposed	95.65	94.69	96.79	97.04	96.98	97.23	95.04	96.52	92.69	97.65	96.02

Algorithm	Specificity										
	MRI 1	MRI 2	MRI 3	MRI 4	MRI 5	MRI 6	MRI 7	MRI8	MRI 9	MRI 10	Avg

*K*-means	86.25	87.25	86.25	89.35	80.25	83.54	86.35	82.47	81.25	82.35	84.53
FCM	87.25	88.54	87.32	89.87	81.25	84.25	87.25	83.24	82.54	83.25	85.47
OTSU	87.98	89.25	88.25	90.25	82.24	85.32	87.96	83.56	83.25	84.25	86.23
PSO	88.25	90.25	89.32	91.55	82.35	85.36	88.35	84.32	84.54	85.24	86.95
BFA	89.95	91.97	94.96	92.78	89.01	91.56	88.56	88.36	90.21	93.32	91.06
GA	91.89	92.25	94.29	92.98	90.89	92.68	92.89	92.25	90.78	92.26	92.31
FLGMM	95.75	92.63	96.85	93.47	91.45	93.47	92.25	94.89	91.65	93.78	93.61
Proposed	96.25	95.99	97.89	97.94	97.87	94.23	96.32	96.69	94.69	98.59	96.64

Algorithm	Segmentation accuracy										
	MRI 1	MRI 2	MRI 3	MRI 4	MRI 5	MRI 6	MRI 7	MRI8	MRI 9	MRI 10	Avg

*K*-means	85.02	86.98	83.43	82.97	81.65	81.98	83.24	83.34	80.12	84.34	83.30
FCM	85.13	87.65	84.31	83.86	83.08	83.57	84.32	84.12	91.34	80.45	84.78
OTSU	87.35	88.32	88.14	88.75	89.52	89.82	87.52	88.45	89.56	84.23	88.16
PSO	89.46	89.01	91.25	91.64	87.25	90.97	88.26	89.67	86.78	87.45	89.17
BFA	90.57	90.14	93.48	92.53	90.75	90.53	89.63	90.32	89.26	91.12	90.83
GA	90.68	91.27	94.26	92.32	91.22	91.45	90.89	92.55	91.54	92.65	91.88
FLGMM	94.97	92.50	95.37	91.11	92.54	94.78	92.78	93.56	91.43	93.78	93.28
Proposed	97.56	94.83	96.34	97.95	96.87	97.98	95.09	96.78	94.33	97.56	96.52

**Table 3:** Performance measures for the proposed method and the existing methods for segmenting Gray Matter (GM) from 10 different MR images.

Algorithm	Sensitivity										
	MRI 1	MRI 2	MRI 3	MRI 4	MRI 5	MRI 6	MRI 7	MRI 8	MRI 9	MRI 10	Avg
*K*-means	84.21	81.56	82.34	81.35	82.54	80.96	82.35	83.25	87.39	84.36	83.03
FCM	85.25	91.36	85.36	84.35	83.25	82.58	84.36	85.36	88.669	87.36	85.78
OTSU	85.35	89.71	88.06	87.72	89.62	89.62	91.72	89.06	89.21	87.24	88.73
PSO	88.28	86.45	89.21	87.26	89.96	88.35	91.25	90.78	89.35	87.98	88.88
BFA	95.67	90.95	89.36	89.23	90.78	89.89	92.35	93.56	89.87	89.25	91.09
GA	96.27	91.90	93.25	92.35	92.35	90.56	93.789	93.25	90.89	90.45	92.50
FLGMM	96.26	92.55	94.78	93.25	93.25	92.35	92.37	95.35	91.23	94.25	93.56
Proposed	98.66	93.12	96.52	95.89	98.23	96.98	97.04	96.79	97.69	94.65	96.55

Algorithm	Specificity										
	MRI 1	MRI 2	MRI 3	MRI 4	MRI 5	MRI 6	MRI 7	MRI8	MRI 9	MRI 10	Avg

*K*-means	86.35	87.36	86.98	89.36	81.25	84.52	86.35	82.98	81.58	83.25	84.99
FCM	87.96	88.69	87.59	90.35	82.32	84.36	87.36	84.35	82.36	84.35	85.96
OTSU	87.69	90.32	89.36	91.25	83.25	86.32	88.25	84.35	84.23	85.32	87.03
PSO	89.32	91.33	90.22	90.25	81.25	85.25	89.35	84.25	84.25	85.96	87.14
BFA	89.25	90.89	94.58	92.89	89.86	90.98	88.26	88.45	90.58	92.89	90.86
GA	92.58	92.89	94.56	92.58	91.25	92.56	91.25	91.56	90.58	91.25	92.10
FLGMM	94.25	91.25	94.25	94.25	92.25	93.25	92.58	93.56	92.25	94.25	93.21
Proposed	97.25	93.88	97.58	97.36	97.25	95.28	96.58	96.25	94.89	99.25	96.55

Algorithm	Segmentation accuracy										
	MRI 1	MRI 2	MRI 3	MRI 4	MRI 5	MRI 6	MRI 7	MRI 8	MRI 9	MRI 10	Avg

*K*-means	85.23	86.35	84.25	82.56	81.25	82.36	84.25	84.35	81.25	84.69	83.65
FCM	85.69	87.89	84.69	84.25	83.69	84.25	85.98	84.25	92.58	81.23	85.45
OTSU	87.98	88.86	88.56	89.56	89.87	88.89	87.89	89.25	90.25	85.02	88.61
PSO	90.25	90.25	91.89	91.58	87.25	90.25	88.96	89.58	86.98	87.58	89.45
BFA	90.58	91.25	92.36	92.69	91.02	90.89	90.25	91.22	90.25	91.69	91.22
GA	90.98	91.35	94.25	92.69	91.75	91.85	90.25	91.78	91.58	92.58	91.90
FLGMM	95.05	92.69	95.86	91.58	92.89	94.25	92.89	93.89	91.25	92.58	93.29
Proposed	97.85	94.98	96.58	98.02	97.25	98.25	95.69	96.84	94.58	97.86	96.79

**Table 4:** Performance measures for the proposed method and the existing methods for segmenting CSF from 10 different MR images.

Algorithm	Sensitivity										
	MRI 1	MRI 2	MRI 3	MRI 4	MRI 5	MRI 6	MRI 7	MRI 8	MRI 9	MRI 10	Avg
*K*-means	85.34	83.25	83.69	82.58	83.56	81.25	83.78	84.25	87.69	85.23	84.06
FCM	86.36	91.89	86.32	85.25	84.25	83.56	85.69	86.45	89.36	88.21	86.73
OTSU	85.98	89.99	89.12	87.56	90.25	90.89	92.45	89.04	89.21	88.25	89.27
PSO	88.69	86.89	90.24	88.20	90.21	89.25	91.78	91.00	90.25	88.25	89.47
BFA	96.25	91.25	90.78	89.21	90.58	90.25	93.01	94.25	88.25	89.12	91.29
GA	96.89	91.99	94.25	93.25	93.56	90.24	92.25	93.25	91.24	91.89	92.88
FLGMM	96.96	92.89	95.14	93.89	93.74	93.58	95.89	95.89	92.58	95.86	94.64
Proposed	99.84	94.25	97.58	96.58	99.21	95.25	97.01	97.21	97.25	94.89	96.90

Algorithm	Specificity										
	MRI 1	MRI 2	MRI 3	MRI 4	MRI 5	MRI 6	MRI 7	MRI 8	MRI 9	MRI 10	Avg

K-means	86.21	87.25	85.25	90.24	81.89	85.28	87.25	83.25	82.54	84.25	85.34
FCM	88.01	89.25	87.98	91.25	83.25	85.25	88.56	85.24	83.25	85.28	86.73
OTSU	87.99	90.89	90.25	91.29	83.24	86.99	88.74	85.20	85.24	85.96	87.57
PSO	90.24	92.14	90.29	90.89	82.14	85.89	90.21	85.21	85.24	86.21	87.84
BFA	90.21	91.25	95.21	93.25	90.21	91.25	89.25	89.24	91.45	93.24	91.45
GA	93.25	93.56	95.24	92.89	92.14	92.89	92.14	92.25	91.25	92.27	92.78
FLGMM	95.89	92.89	95.86	95.76	92.89	93.56	92.78	93.86	92.86	94.78	94.11
Proposed	97.85	94.85	97.89	97.86	97.58	96.24	95.21	96.57	94.85	99.14	96.80

Algorithm	Segmentation accuracy										
	MRI 1	MRI 2	MRI 3	MRI 4	MRI 5	MRI 6	MRI 7	MRI 8	MRI 9	MRI 10	Avg

*K*-means	85.78	86.59	85.24	83.25	82.12	86.25	85.24	84.25	82.21	85.25	84.61
FCM	85.96	87.25	84.89	84.56	83.96	84.58	85.99	84.89	92.56	82.54	85.71
OTSU	87.96	89.56	88.21	88.56	88.56	89.12	87.88	89.59	90.78	85.89	88.61
PSO	90.45	91.24	91.99	92.12	88.21	91.25	89.25	90.21	87.21	87.89	89.98
BFA	90.24	91.53	92.01	92.58	91.56	91.89	90.21	91.25	91.88	91.54	91.46
GA	90.99	92.45	95.89	93.21	92.14	92.45	91.26	92.79	91.89	92.96	92.60
FLGMM	95.86	92.99	95.99	91.89	92.56	94.58	93.25	96.89	93.58	93.12	94.07
Proposed	97.58	95.01	96.89	98.25	97.14	98.45	95.25	96.99	95.84	98.21	96.96
